# Clade-specific adaptation and global spread of *Staphylococcus aureus* ST188 with emergence of a multidrug-resistant MRSA sublineage

**DOI:** 10.1128/msystems.00848-25

**Published:** 2025-09-24

**Authors:** Deru Lei, Xu Dong, Ting Yang, Ye Jin, Wangxiao Zhou

**Affiliations:** 1 Clinical Laboratory Center, The Second Affiliated Hospital & Yuying Children's Hospital of Wenzhou Medical University 26453 , Wenzhou, Zhejiang, People’s Republic of China; 2 State Key Laboratory for Diagnosis and Treatment of Infectious Diseases, National Clinical Research Center for Infectious Diseases, Collaborative Innovation Center for Diagnosis and Treatment of Infectious Diseases, The First Affiliated Hospital, Zhejiang University School of Medicine 26441, Hangzhou, Zhejiang, People’s Republic of China; 3 Department of General Intensive Care Unit, The Second Affiliated Hospital of Zhejiang University School of Medicine 26441, Hangzhou, Zhejiang, People's Republic of China; 4 Key Laboratory of Early Warning and Intervention of Multiple Organ Failure, China National Ministry of Education, Hangzhou, Zhejiang, People's Republic of China; University of Birmingham, Birmingham, United Kingdom

**Keywords:** *Staphylococcus aureus*, sequence type 188, genomic analysis, antimicrobial resistance, virulence, mobile genetic elements

## Abstract

*Staphylococcus aureus* sequence type (ST) 188 is a globally distributed lineage frequently associated with colonization and bloodstream infection in both humans and animals, yet its evolutionary dynamics and genomic adaptations remain poorly understood. In this study, we conducted a comprehensive genomic analysis of 808 ST188 isolates collected from 24 countries between 2004 and 2023. Phylogenetic reconstruction identified seven clades, with clades I and VII showing independent clonal expansions in China. Frequent cross-regional, international, and cross-host transmission events were observed, supporting the emergence of ST188 as a host-generalist lineage. A distinct methicillin-resistant *S. aureus* subclade within clade VI likely emerged from a methicillin-susceptible ancestor through the acquisition of SCC*mec* IVa. This event was accompanied by co-acquisition of resistance transposon Tn*6636* and fluoroquinolone-resistance mutations, alongside truncation of the adhesion-related gene *sraP* and loss of the serine protease genes *splDE*. Preliminary phenotypic assays confirmed reduced adhesion and colonization in clade VI isolates. Comparative analysis revealed clade-specific patterns of mobile genetic elements, including vertical inheritance of SaPI1 and SaPI2 in the Chinese subclade of clade VII. In contrast, the novel prophage φST188-1, found exclusively in clade VII isolates, appeared to have been independently acquired. However, accessory genome variation across clades was limited, and the overall population structure was primarily shaped by core genome single-nucleotide polymorphisms. These findings provide a detailed view of the evolution and adaptation of ST188, underscore the role of clade-specific resistance and virulence patterns, and highlight the importance of continued genomic surveillance of this emerging lineage.

**IMPORTANCE:**

The global emergence of *Staphylococcus aureus* ST188 poses new challenges to public health due to its ability to infect both humans and animals and spread across regions and continents. Despite its growing prevalence, little has been known about its evolutionary history and dissemination patterns. In this study, we analyzed 808 ST188 genomes from 24 countries and found evidence of frequent cross-regional and cross-host transmission. Two major clades, showing clear clonal expansion, were dominated by isolates from China. We also identified a newly emerged methicillin-resistant subclade likely derived from a methicillin-susceptible ancestor, characterized by the acquisition of SCC*mec* IVa, multiple resistance genes, and fluoroquinolone-resistance mutations. This subclade exhibited reduced adhesion and colonization capacity due to structural loss of key virulence genes. These findings provide new insights into the clade-specific adaptation and global spread of ST188 and underscore the need for genomic surveillance of multidrug-resistant *S. aureus* emerging from traditionally susceptible lineages.

## INTRODUCTION

*Staphylococcus aureus* is a common human colonizer and one of the most significant opportunistic bacterial pathogens worldwide, frequently associated with severe invasive infections (1). As a multi-host opportunistic pathogen with a long evolutionary history, *S. aureus* persistently colonizes approximately 30% of the human anterior nares and may also be found on the skin, in the throat, axillae, and perineal regions (2). Although colonization is typically asymptomatic, it substantially increases the risk of infection. Studies have shown that in up to 80% of infection cases, the infecting strain is genetically identical to the colonizing strain (3). When the skin or mucosal barriers are disrupted due to chronic skin diseases, wounds, or surgical procedures, *S. aureus* can invade underlying tissues or enter the bloodstream (4). Patients with invasive medical devices, such as peripheral or central venous catheters, or those who are immunocompromised, are particularly vulnerable to *S. aureus* infections (1).

The ST188 lineage exhibits a high colonization rate in the nasal cavities of both healthy individuals and animals and is capable of causing infections in various animal hosts (5, 6). Notably, it is one of the most common methicillin-susceptible *S. aureus* (MSSA) lineages associated with bovine mastitis (7). Wang et al. (8) hypothesized that ST188 acquired the ability to colonize diverse hosts through the upregulation of factors involved in bacterial adhesion and biofilm formation. In addition, atopic dermatitis (AD), one of the most prevalent inflammatory skin disorders, has shown a marked increase in incidence over the past decade, contributing to a significant global disease burden (9). Several studies have identified ST188 as the most frequently detected sequence type (ST) on AD-affected skin, with its isolation rate and relative abundance significantly increasing with disease severity (10, 11). Moreover, ST188 predominates among isolates from Asian AD patients and infants prior to AD onset (12). More alarmingly, recent epidemiological data indicate that ST188 has become the most prevalent MSSA lineage responsible for adult bloodstream infections in China (13). Although ST188 methicillin-resistant *S. aureus* (MRSA) remains relatively rare, it has been detected across geographically diverse regions, including North America, Asia, and Australia, according to genomic surveillance data from Pathogenwatch (https://pathogen.watch/collection/ye8hoxm743f7-st188), reflecting its global distribution. This is particularly concerning given that MSSA can serve as a reservoir for the emergence of MRSA through the acquisition of the staphylococcal cassette chromosome *mec* (SCC*mec*) element (14). Despite these insights, most previous studies have focused on the epidemiological characteristics of ST188, and a comprehensive understanding of its genomic evolution and genotypic characteristics remains lacking.

This study performed a comprehensive genomic analysis of 808 globally collected *S. aureus* ST188 isolates from 2004 to 2023, aiming to elucidate their population structure, evolutionary divergence, and key genomic traits. Phylogenetic reconstruction was used to trace clade-level divergence and cross-regional or cross-host transmission. By comparing antimicrobial resistance (AMR) genes, virulence factors, and mobile genetic elements (MGEs) across clades, and analyzing accessory genome content alongside core single-nucleotide polymorphism (SNP) variation, this study reveals the evolutionary dynamics and lineage-specific adaptations of ST188. These findings advance our understanding of its diversification, including the emergence of MRSA subclades, and provide a valuable framework for future genomic surveillance and functional studies.

## MATERIALS AND METHODS

### Collection of bacterial isolates

As of July 2024, a total of 85,893 *S*. *aureus* genome assemblies were retrieved from the NCBI GenBank database. STs were assigned using the MLST v2.0 web tool (https://cge.food.dtu.dk/services/MLST/), and only ST188 isolates with available geographic and collection date metadata were included for further analysis. In addition, 29 ST188 isolates (2 MRSA and 27 MSSA) were prospectively collected between 2021 and 2023 from blood, urine, wound/pus, and respiratory specimens of patients under routine clinical care at the Second Affiliated Hospital & Yuying Children’s Hospital of Wenzhou Medical University. According to the Centers for Disease Control and Prevention’s definition, all 29 isolates were classified as community-associated. Antimicrobial susceptibility testing for these isolates was performed using the VITEK-2 system (bioMérieux, France). In total, 808 ST188 isolates collected from 24 countries across five continents between 2004 and 2023 were included in this study (Table S1).

### Whole-genome sequencing and genomic analysis of ST188 isolates

Genomic DNA from the 29 ST188 isolates was extracted using the Ezup Column Bacteria Genomic DNA Purification Kit (Sangon Biotech, Shanghai, China). Whole-genome sequencing was performed on the Illumina HiSeq X Ten platform (San Diego, CA, USA) with a 2  ×  150  bp read length. Raw reads were trimmed and quality-filtered using fastp v0.23.2 (15), and *de novo* assembly was carried out with SPAdes v3.13.0 (16). Long-read sequencing of the representative isolate M2024 was performed using the Oxford Nanopore platform (Oxford Nanopore Technologies, Oxford, UK). Hybrid genome assembly combining long reads and Illumina short reads was conducted using Unicycler v0.5.0 (17) to generate a complete genome sequence. SCC*mec* and *spa* typing were performed using the web-based tools SCCmecFinder (https://cge.food.dtu.dk/services/SCCmecFinder) and SpaTyper (https://cge.food.dtu.dk/services/spaTyper), respectively. Genome annotation was conducted using DFAST v1.2.18 (18). Pan-genome analysis of ST188 isolates was conducted using Panaroo v1.3.0 (19) under the strict mode, defining accessory genes as those present in less than 100% of the isolates. Non-linear dimensionality reduction of the accessory gene content matrix was performed using the Rtsne package in R.

### Phylogenetic analysis and Bayesian evolutionary analysis

Core genome alignment of the 808 ST188 isolates was performed against the reference genome M2024 (GenBank accession no. CP195249.1) using Snippy v4.6.0 (https://github.com/tseemann/snippy). Recombinant regions were identified and removed using Gubbins v2.4.1 (20) prior to phylogenetic analysis. A maximum likelihood phylogenetic tree was constructed with RAxML v8.2.12 (21)  under the GTRGAMMAX model with 1,000 bootstrap replicates. The genome of *S. aureus* MW2 (ST1, accession no. NC_003923) was used as an outgroup. Temporal phylogenetic reconstruction was conducted using BactDating v1.1 (22) under the mixedcarc model with 200 million MCMC iterations. Convergence was confirmed by effective sample sizes (ESS) > 200 for all parameters. Final phylogenetic trees were visualized using ggtree v3.7.1.003, annotated with corresponding metadata. Clonal transmission clusters with a core-genome SNP distance <24 were visualized using Cytoscape v3.8.2 (23). Non-linear dimensionality reduction of the core SNP matrix was performed using the Rtsne package in R.

### Identification of AMR genes, virulence genes, and MGEs among ST188 isolates

AMR genes were identified using AMRFinderPlus v3.11.20 (24), while virulence factors were predicted with ABRicate v1.0.0 (https://github.com/tseemann/abricate) based on the VFDB database, applying thresholds of ≥90% sequence identity and ≥90% coverage. Pathogenicity islands and prophages were detected via BLASTN (https://blast.ncbi.nlm.nih.gov/Blast.cgi) using publicly available *S. aureus* MGE sequences as references, with a minimum identity and query coverage of 85%. Linear comparisons of MGEs were visualized using the gggenomes R package (v0.9.5.9000, https://github.com/thackl/gggenomes).

### Mice nasal colonization model

For each tested strain, a group of five mice (*n* = 5) was intranasally inoculated with 10  µL of sterile PBS containing *S. aureus* cells adjusted to a 0.5 McFarland turbidity standard. After a 5-day colonization period under standard housing conditions, mice were euthanized, and nasal tissues were aseptically collected and homogenized. Bacterial colonization levels were quantified by plating 200  µL of serially diluted homogenates onto TSA plates.

### Adhesion assay

The adhesion capacity of strains from different clades was evaluated as previously described (8). Overnight bacterial cultures were adjusted to an OD_600_ of 0.5 and then incubated with A549 human alveolar epithelial cells at a 37 °C incubator with 5% CO_2_. After 2 h of co-culture, non-adherent bacteria were removed by washing with PBS, and the epithelial cells were subsequently lysed using 0.1% sodium deoxycholate to release both surface-adherent and internalized bacteria for enumeration.

### Cytokine determination

Murine macrophage RAW264.7 cells were cultured in high-glucose DMEM (Gibco, Thermo Fisher Scientific, Waltham, MA, USA), supplemented with 10% fetal bovine serum (FBS; Sigma-Aldrich, St. Louis, MO, USA) and 1% penicillin-streptomycin (Thermo Fisher Scientific). The cells were seeded into sterile six-well culture plates (Corning, NY, USA) at a density of 3 × 10^5^ cells/mL and incubated for 24 h at a 37°C incubator with 5% CO_2_. Bacterial cultures were adjusted to 1.0 × 10^9^ CFU/mL in sterile PBS. A 10 µL volume of this suspension was added to each well to achieve a multiplicity of infection of 30. Co-culture was carried out at 37°C for 6 h. After incubation, cell culture supernatants were harvested and centrifuged at 2,000 × *g* for 20 min at 4°C to eliminate residual debris. The levels of IL-6 and TNF-α in the cleared supernatants were measured using enzyme-linked immunosorbent assay kits, according to the manufacturer’s protocols (R&D Systems, USA).

### Whole-blood killing assay

*S. aureus* strains were cultured in TSB until the post-exponential phase. Bacterial suspensions were then prepared at approximately 2.5 × 10^8^ CFU and added to fresh heparinized human whole blood. The mixture was incubated at 37 °C for 1 h under continuous rotation. Following incubation, samples were serially diluted and plated on TSA to determine viable bacterial counts.

### Statistical analysis

All data processing and statistical analyses were performed using R software (version 4.2). Categorical variables were summarized as counts and percentages, with group comparisons assessed using the chi-squared test or Fisher’s exact test, as appropriate. Continuous variables were expressed as either mean ± standard deviation, and between-group differences were evaluated using the Wilcoxon rank-sum test. A two-tailed *P* value < 0.05 was considered statistically significant.

## RESULTS

### Geographic distribution and evolutionary trajectory of the *S. aureus* ST188 isolates

**Fig 1:**
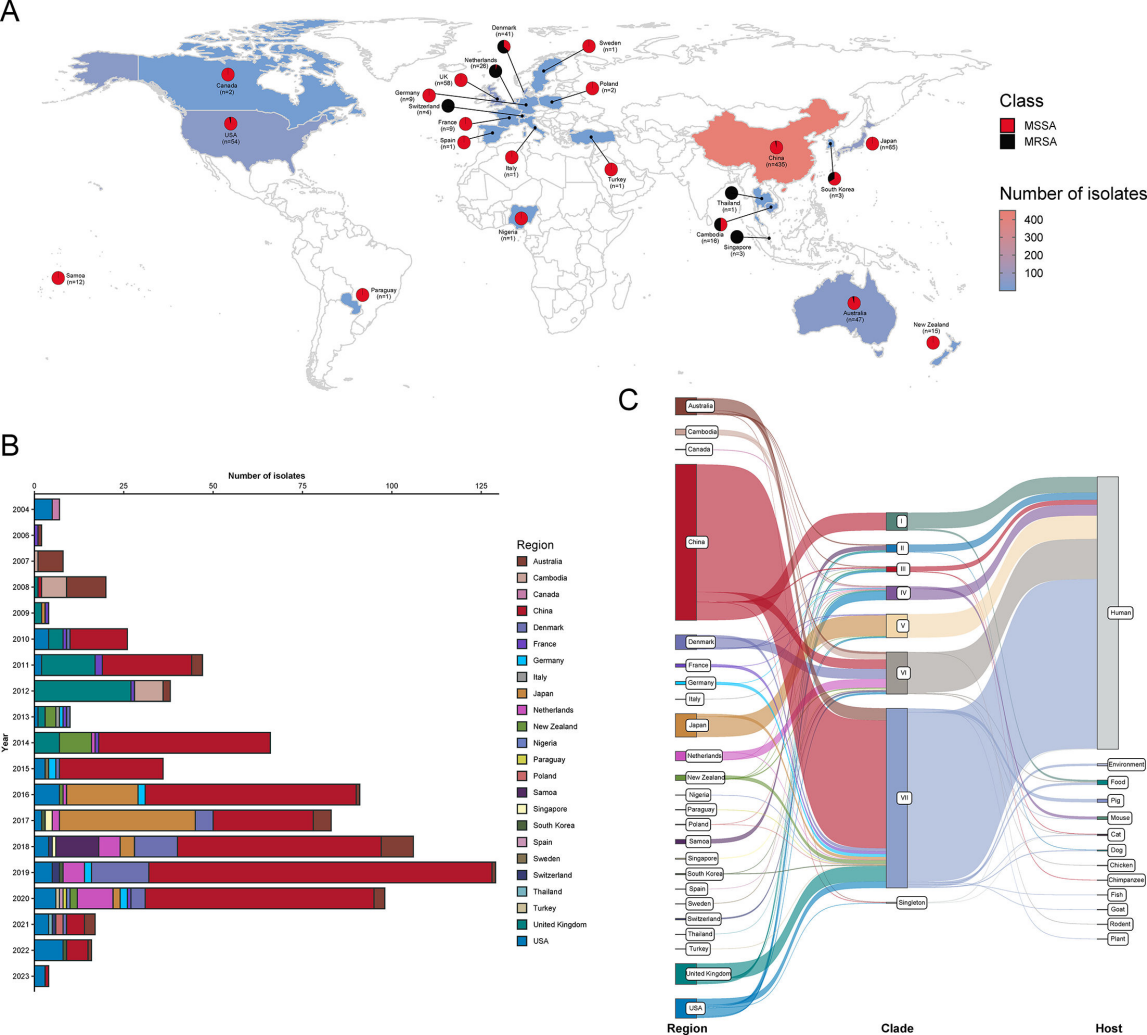
Relationships among 808 ST188 isolates in this study. (**A**) Geographic locations of all ST188 isolates included in this study. Red and black dots represent MSSA and MRSA, respectively. Countries with ST188 isolates are highlighted with background shading. (**B**) Temporal and geographic distribution of ST188 isolates from 2004 to 2023. The bar plot displays the annual number of isolates, with bar colors corresponding to different countries. (**C**) Sankey diagram showing the relationships among geographic regions, phylogenetic clades, and host sources of the ST188 isolates. Data are available at https://microreact.org/project/aUNS9EtTXiHX6LUTUw3ckY-clade-specific-adaptation-and-global-spread-of-staphylococcus-aureus-st188-with-emergence-of-a-multidrug-resistant-mrsa-sublineage.

To comprehensively characterize the global molecular evolutionary patterns of *S. aureus* ST188, we included whole-genome assemblies from 808 ST188 isolates collected between 2004 and 2023 from 24 countries across Asia, Europe, the Americas, Africa, and Oceania. Of these, 29 community-acquired isolates originated from our collection. The geographical distribution of ST188 is shown in Fig. 1A, with the highest numbers of isolates found in China (53.84%, 435/808), Japan (8.04%, 65/808), the United Kingdom (7.18%, 58/808), the United States (6.68%, 54/808), and Australia (5.82%, 47/808). Notably, isolates from China have increasingly dominated the data set since 2010 (Fig. 1B). Although the majority (89.48%, 723/808) of isolates were MSSA, a total of 85 MRSA isolates were identified across 10 countries spanning four continents (including 13 isolates from China), indicating significant geographic diversity among ST188 MRSA strains (Fig. 1A). Importantly, the 435 Chinese isolates were collected from 21 different provinces (Fig. S1), covering approximately 76.22% of the country’s population (around 1.1 billion people), suggesting wide dissemination of this lineage within China. Detailed information on these ST188 isolates is listed in Table S1.

**Fig 2:**
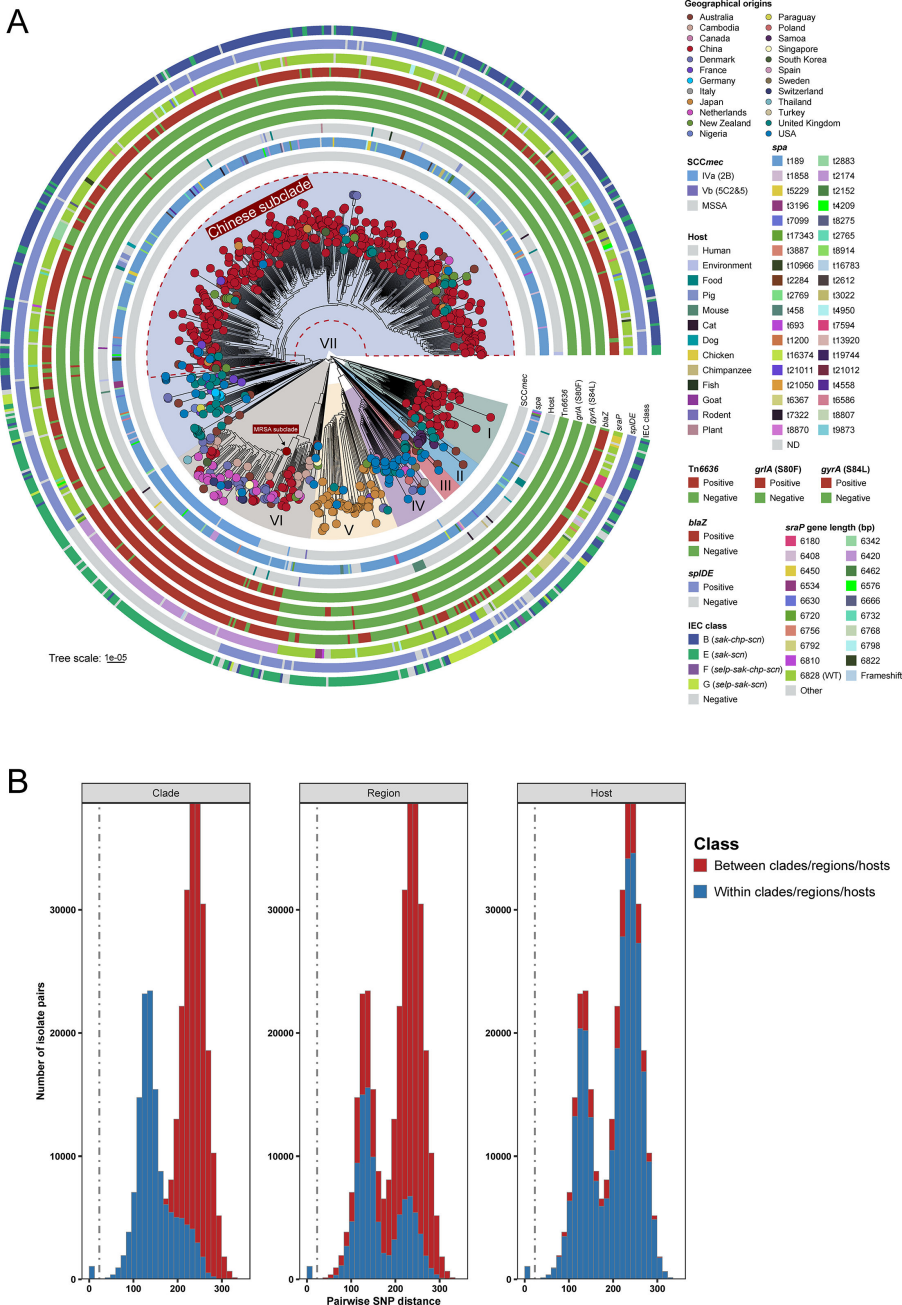
Phylogenetic analysis of 808 ST188 isolates. (**A**) Maximum-likelihood phylogenetic tree based on core genome SNPs. From inner to outer rings: SCC*mec* type, *spa* type, host origin, presence of Tn*6636*, quinolone resistance-determining region (QRDR) mutations (*grlA* and *gyrA*), presence of *blaZ*, length of *sraP*, presence of *splDE*, and IEC type. Tip colors correspond to the country of isolation. (**B**) Histogram of pairwise SNP distances among all ST188 isolates. The vertical gray dashed line indicates the 23-SNP threshold used to define potential transmission events.

Phylogenomic analysis based on 36,472 non-recombinant core SNPs indicated that the most recent common ancestor of all ST188 isolates emerged around 1954 (95% highest posterior density: 1945–1962; Fig. S2), subsequently diverging shortly thereafter into seven major phylogenetic clades (I–VII), with estimated divergence times ranging from 1958 to 1977 (Fig. 2A; Fig. S2). These findings suggest that global dissemination of ST188 has been ongoing for at least several decades. Furthermore, the phylogenetic analyses demonstrated that Chinese ST188 isolates originated from multiple lineages: except for sporadic isolates in clades III and VI, all isolates from clade I and up to 71.20% of isolates from clade VII were collected from China, displaying local expansion. Additionally, isolates within clade IV predominantly originated from the United States (63.16%), whereas those within clade V primarily originated from Japan (87.69%). Other clades lacked distinct geographical distributions (Fig. 1C and 2A). Notably, apart from three ST188 MRSA isolates scattered across the phylogenetic tree, the majority of MRSA isolates clustered distinctly in clade VI, likely due to a single introduction event of SCC*mec* IVa around 1996, forming a unique MRSA subclade (Fig. 2A; Fig. S2 ). The close phylogenetic association of these MRSA isolates with basal MSSA isolates in clade VI further suggests that ST188 MRSA likely emerged from ancestral MSSA strains. In addition, although genotypic *spa* typing identified a total of 38 *spa* types among the 808 ST188 isolates, *spa* t189 was predominant across all clades, with prevalence ranging from 83.67% to 93.33%.

Although some isolates from the same country formed tight clusters on the phylogenetic tree, isolates from different countries or even continents clustered together in all clades except for clade I (Fig. 2A), with no clear geographic boundaries among isolates from different Chinese provinces in China (Fig. S1A). Interestingly, while the vast majority of isolates were human-derived, the remaining 49 isolates originated from 12 different host sources, covering food (*n* = 13), animals (*n* = 29), the environment (*n* = 6), and plants (*n* = 1). Phylogenetic analysis revealed that these non-human isolates were dispersed throughout the tree and closely related to neighboring human-derived isolates (Fig. 2A). Furthermore, pairwise SNP differences indicated that the core genome SNP variation between isolates from different clades ranged from 155 to 347 (median = 241), suggesting substantial genetic divergence among the major clades (Fig. 2B). Using a threshold of fewer than 24 SNP differences as indicative of possible involvement in clonal transmission events for *S. aureus* (25), we identified 78 independent clonal transmission events across 11 countries, including international transmissions between the United States and Canada, and transmission events involving pigs, farm workers, and the environment on a farm in China. Additionally, the 31 small-scale transmission events identified in China spanned 14 different provinces, including three inter-provincial transmission events.

### Distributions of AMR genes in the ST188 lineage

**Fig 3:**
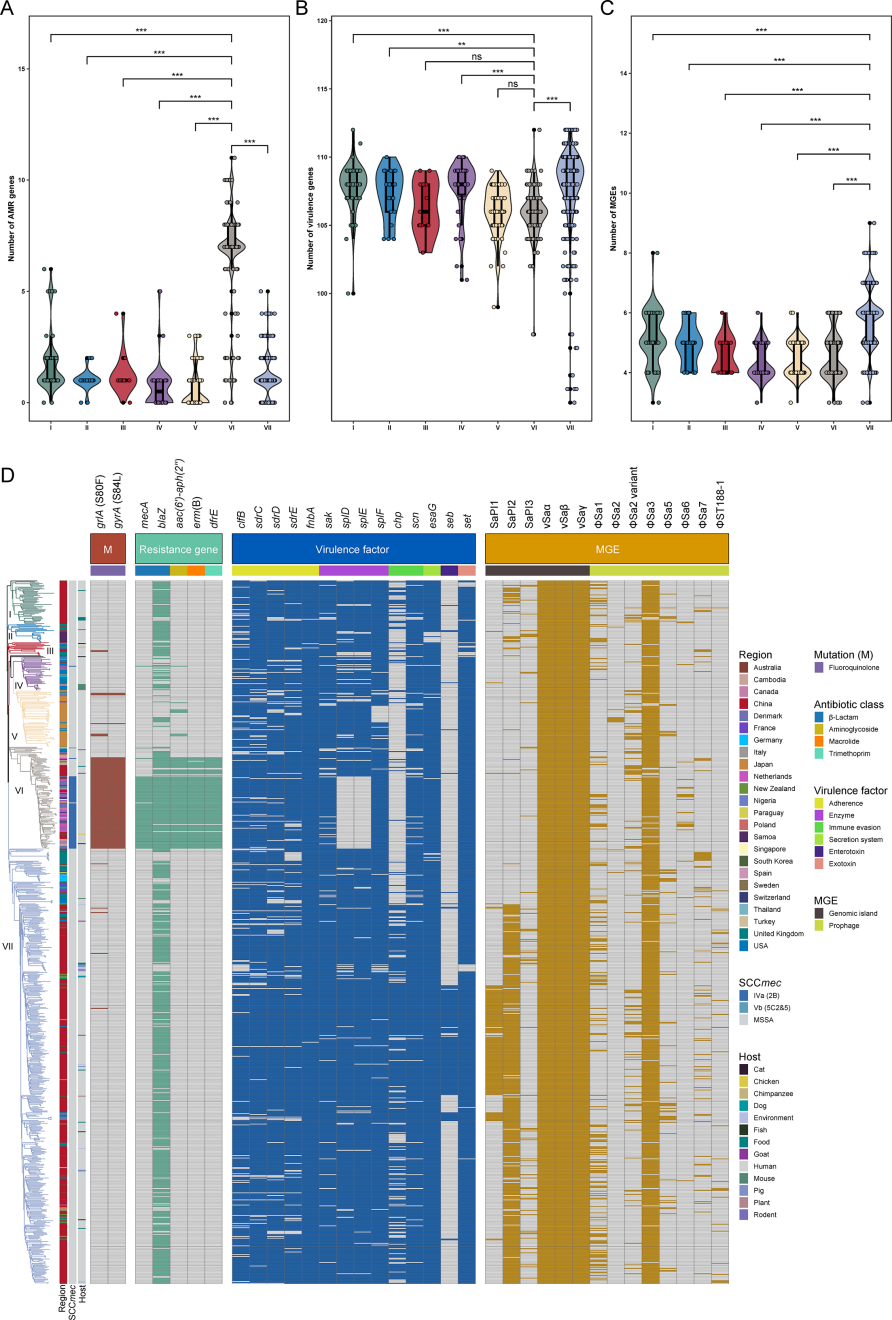
Distribution of AMR genes, virulence genes, and MGEs in ST188 isolates. (**A–C**) Distribution of AMR genes (**A**), virulence genes (**B**) and MGEs (**C**) across isolates from various clades. **, *P* < 0.01; ***, *P* < 0.001. ns, no significant difference between groups. (**D**) Heatmap showing the distribution of chromosomal mutations, AMR genes, virulence genes, and MGEs across ST188 isolates. The presence of each element is represented by color-coded boxes. For chromosomal mutations, AMR and virulence genes, only specific genes present in more than 10% of isolates were retained, and genes present in >90% of isolates across all clades were omitted to highlight differentially prevalent virulence traits.

To investigate the AMR profiles of global ST188 isolates, we assessed the distribution of resistance genes and chromosomal mutations across different phylogenetic clades. In total, 38 resistance genes conferring resistance to 15 classes of antibiotics were identified (Fig. S3). Notably, isolates within clade VI harbored a median of seven resistance genes, significantly higher than those in all other clades (median range: 0–1; Fig. 3A). Further analysis showed that clade VI isolates frequently carried the aminoglycoside resistance gene *aac(6′)-aph(2″)* (84.62%), the macrolide resistance gene *erm*(B) (83.76%), and the trimethoprim resistance gene *dfrE* (84.62%), all of which were significantly more prevalent than in other clades. Notably, these genes are co-located within the composite transposon Tn*6636*. In addition, fluoroquinolone resistance-associated mutations *grlA* S80F (89.74%) and *gyrA* S84L (89.74%) were also highly enriched in clade VI (Fig. 3D). These resistant isolates primarily belonged to the MRSA subclade within clade VI and its closely related MSSA counterparts (Fig. 2A and 3D), further supporting the hypothesis that ST188 MRSA carrying SCC*mec* IVa may have evolved from methicillin-susceptible ancestral ST188 strains.

The β-lactamase gene *blaZ* was found in over 84% of isolates in all clades except clades IV and V, where its prevalence dropped to 50% and 13.85%, respectively. Specifically, 63.15% of *blaZ*-negative isolates in clade IV were from the United States, while 87.72% of the *blaZ*-negative isolates in clade V were from Japan, suggesting a potential trend toward penicillin susceptibility in specific lineages. Furthermore, more than 24% of isolates in clade I carried the macrolide resistance gene *erm*(C) and the trimethoprim resistance gene *dfrG*, both of which had a prevalence of less than 12% in other clades. Additionally, the remaining resistance genes were sporadically distributed across the ST188 lineage, with overall low prevalence (<16%) in all clades (Fig. S3).

We also evaluated the *in vitro* susceptibility of 29 Chinese ST188 isolates from our collection to 15 commonly used antibiotics (Table S2). The results demonstrated a strong correlation between predicted resistance genotypes and observed antimicrobial susceptibility patterns.

### Comparison of virulence genes in ST188 clades

To assess the pathogenic potential of isolates across different phylogenetic clades, we analyzed the distribution of virulence factors in the global ST188 lineage using the VFDB database. A total of 127 virulence genes were identified across all isolates, of which 96 (75.59%) were present in over 90% of isolates from all clades. These genes covered functions related to iron uptake, immune evasion, adhesion, enzymes, secretion systems, hemolysins, phenol-soluble modulins, enterotoxins, and leukocidins. Notably, clade VI isolates, which exhibited the highest level of AMR, carried a significantly lower number of virulence genes (median = 106) compared to isolates from clades I, II, IV, and VII (medians = 108, 108, 108, and 109, respectively), but similar to those from clades III and V (both median = 106; Fig. 3B).

Specifically, as shown in Fig. 3D and Fig. S3, the serine protease genes *splD* and *splE* were detected in only 25.64% of clade VI isolates, markedly lower than in other clades (all >76.19%), with these gene losses exclusively observed in the MRSA subclade of VI. The enterotoxin gene *seb* was mainly restricted to clade VII (27.4%), and 85.40% of *seb*-positive isolates were from China. In contrast, *seb* was rarely detected in other clades, with prevalence ranging from 0% to 9.52%. Although the enterotoxin genes *sec* and *sell*, and the toxic shock syndrome toxin gene *tsst-1* were generally uncommon among the ST188 lineage, they were identified in 42 Chinese isolates, 88.10% of which belonged to clade VII. Interestingly, while nearly all ST188 isolates carried genes encoding the immune evasion cluster (IEC) (762/808, 94.31%), the distribution of IEC types varied by clade (Fig. 2A and 3D). IEC type E (*sak-scn*) predominated in clades II, III, V, and VI, whereas type G (*selp-sak-scn*) was dominant in clade IV. In contrast, IEC type B (*sak-chp-scn*), which includes the chemotaxis inhibitory protein gene *chp*, was prevalent in clades I and VII, accounting for 44.90% (22/49) and 69.80% (349/500) of isolates, respectively. Importantly, all *chp*-positive isolates in clade I and 79.42% in clade VII were of Chinese origin. In addition, the Panton-Valentine leukocidin (PVL) genes and exfoliative toxin gene *eta* could be detected in six and two ST188 strains, respectively.

** **Given the strong adhesive capacity associated with ST188 (8), we conducted a detailed analysis of adhesion-related genes across different phylogenetic clades. The results showed that, except for clade VI, the majority of isolates in other clades carried the wild-type *sraP* gene (prevalence ranging from 57.14% to 86.67%), which encodes a protein composed of an N-terminal signal peptide (SP, 1–90 aa), a short serine-rich region (SRR1, 91–244 aa), a non-repeat region (NRR, 245–751 aa) responsible for ligand binding, a long serine-rich region (SRR2, 752–2,225 aa), and a C-terminal cell wall anchoring motif (CW, 2,226–2,275 aa). In contrast, 88.03% (103/117) of clade VI isolates exhibited an internal deletion of 136 amino acids within the SRR2 domain (Fig. 2A; Fig. S4). Such *sraP* truncations were exclusively observed in the MRSA subclade and its closely related MSSA isolates in clade VI, suggesting that these structural alterations may reduce the adhesive capacity of clade VI isolates.

**Fig 4:**
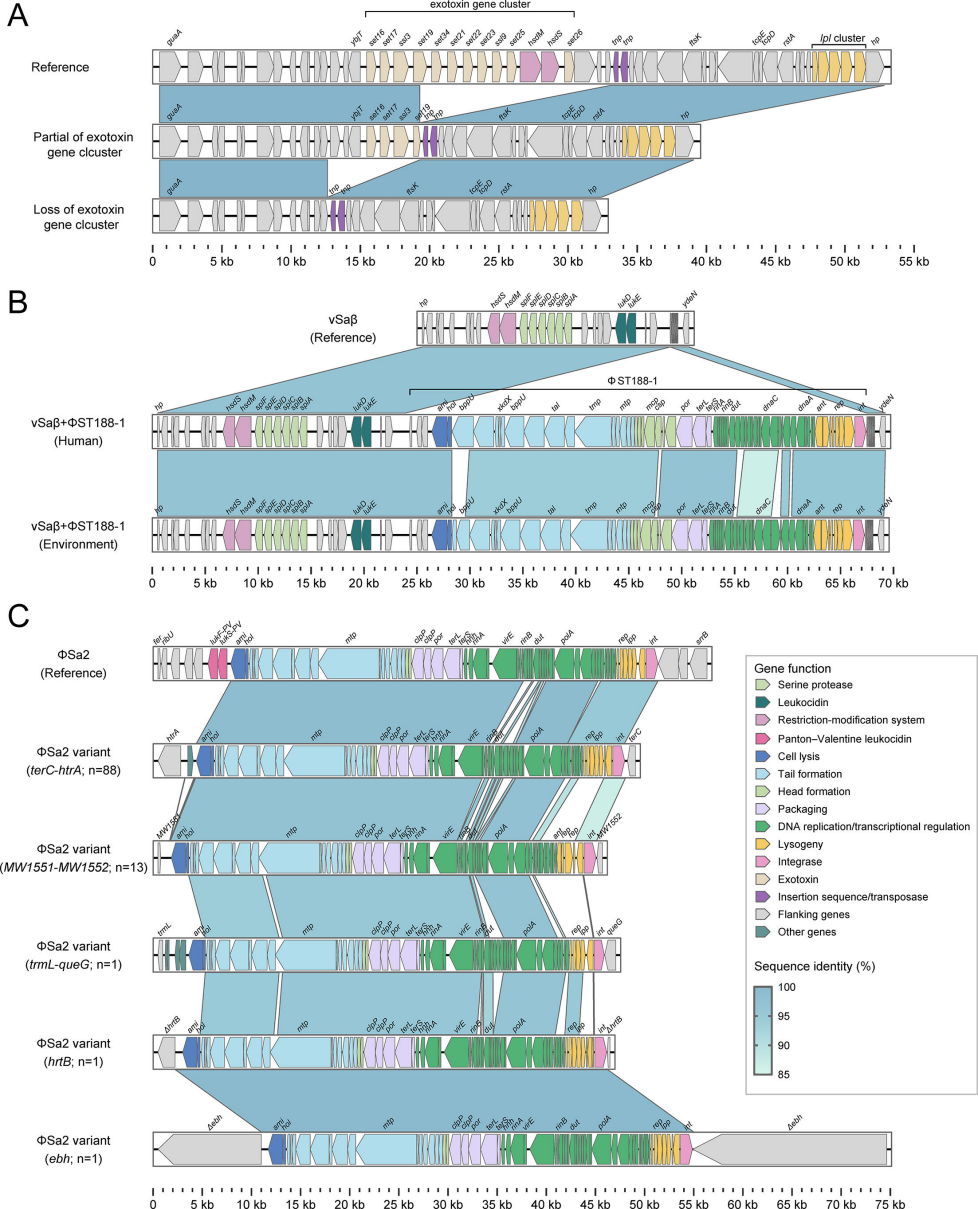
Comparison of genomic islands and prophages among ST188 isolates. Comparison of vSaα (**A**), vSaβ/φST188-1 (**B**), and φSa2 (**C**) between ST188 strains. Genes are indicated by arrowed boxes and colored based on gene function classification.

 Due to the limited number of isolates in our collection, including 4 from clade I, 2 from clade VI, and 22 from clade VII, we selected all available isolates from clades I and VI, along with 4 randomly chosen isolates from clade VII, for preliminary virulence assessment. As shown in Fig. S5A and B, clade VI isolates exhibited significantly lower nasal colonization capacity and epithelial cell adhesion ability in mice compared to isolates from clades I and VII (*P* < 0.001), providing initial support for our hypothesis regarding reduced adhesion of clade VI. Furthermore, comparison of pathogenicity among the clades revealed that clade VI isolates induced lower expression levels of inflammatory cytokines (IL-6 and TNF-α) and showed reduced survival rates in whole-blood killing assays relative to the other two clades. In contrast, clade VII isolates exhibited the highest overall virulence potential (Fig. S5C through E).

### Comparison of MGE profiles among ST188 clades

Horizontal gene transfer plays a critical role in shaping the genome architecture of *S. aureus* by facilitating the exchange of genetic material, particularly MGEs. To further explore this aspect, we investigated the distribution of large MGEs including *S. aureus* pathogenicity islands (SaPIs), genomic islands, and prophages across all ST188 isolates. The results showed that all isolates carried the genomic islands vSaα, vSaβ, and vSaγ, and that more than 89% of isolates in each clade harbored the prophage φSa3. As shown in Fig. 3C, clade VII isolates contained significantly more MGEs than those in other clades, with an average of 5.77 MGEs per isolate. This higher MGE load was primarily due to the elevated prevalence of *seb*-harboring SaPI1, found in 27.20% (136/500) of isolates, and SaPI2, found in 81.00% (405/500). Isolates harboring either SaPI1 or SaPI2 in the Chinese subclade of clade VII formed a monophyletic cluster, which suggests that these elements were mainly spread through vertical transmission within this lineage. Although SaPI3, which encodes *sec*, *sell*, and *tsst-1*, along with prophages φSa5 and φSa7, was detected at low overall frequencies, the positive isolates were dispersed throughout the Chinese subclade of clade VII (Fig. 3D; Fig. S3).

While all ST188 clades carried the genomic islands vSaα and vSaβ, we observed clade-specific structural variation. In clade III, isolates frequently showed loss of the enterotoxin gene cluster within vSaβ (Fig. 4A), with only 46.67% (7/15) strains retaining the intact gene cluster. In addition, a novel prophage, designated φST188-1, was identified within the vSaβ of 28 clade VII isolates, including 26 from China. No known virulence genes were found within this element. Structural analysis indicated that φST188-1 shared modular organization with typical *S. aureus* prophages, consisting of the lysis module, structural module (tail, head, and packaging), DNA replication and transcriptional regulation module, and lysogeny module (Fig. 4B). Notably, highly similar φST188-1 elements were detected in both human-derived and environment-derived isolates from clade VII, suggesting that the prophage may be capable of transmission between different host niches. A BLASTN search against the NCBI nucleotide database showed that φST188-1 shared sequence similarity with those from five publicly available *S. aureus* genomes, with alignment coverage above 78% and nucleotide identity above 95% (Fig. S6A). However, phylogenetic analysis indicated that φST188-1 elements in clade VII likely originated from a single ancestral acquisition (Fig. S6B).

In addition, we identified 104 ST188 isolates carrying φSa2 that was not integrated at the conventional site downstream of the *srrB* gene. Instead, integration occurred at alternative genomic loci, including between the membrane protein-encoding gene *terC* and the serine protease gene *htrA* (*n* = 88), between the membrane protein gene *MW1551* and the predicted enterotoxin gene *MW1552* (*n* = 13), between the tRNA methyltransferase gene *trmL* and the epoxyqueuosine reductase gene *queG*, and within the coding regions of the hemin transport permease gene *hrtB* and the adhesion gene *ebh*. Comparative analysis revealed that sequence differences among these φSa2 variants were primarily located within the DNA replication and transcription regulation modules, as well as in the lysogeny modules (Fig. 4C). Further analysis of φSa2 variants integrated between *terC* and *htrA* showed that positive isolates were sporadically distributed across the entire clades V (*n* = 18), VI (*n* = 17), and VII (*n* = 49). This suggests that these φSa2 variants may have been acquired independently by isolates from different clades. Notably, all φSa2-positive isolates in clade VII were of Chinese origin.

### Core SNPs significantly drove the evolution of the ST188 lineage

**Fig 5:**
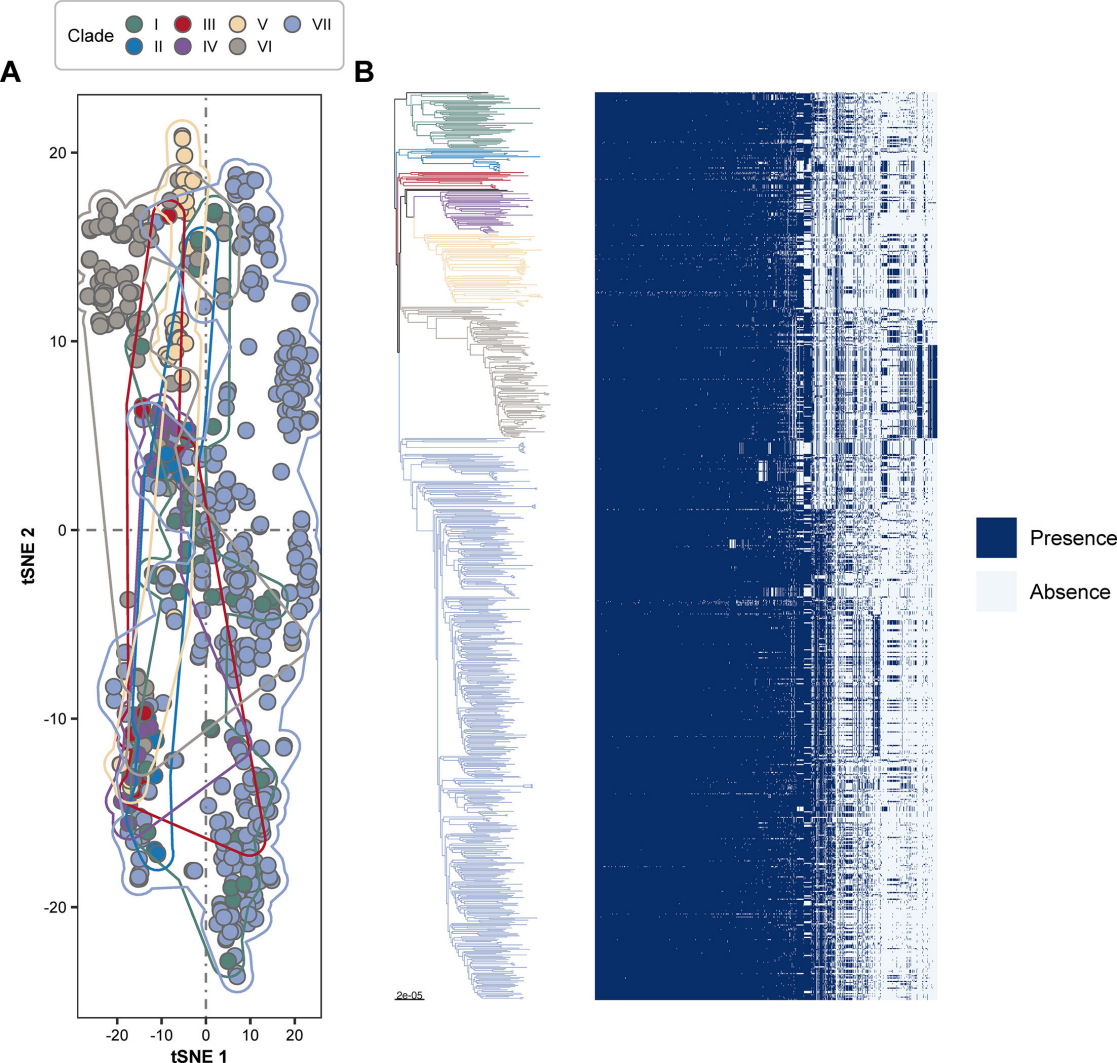
Accessory-genome diversity of ST188 isolates. (**A**) t-SNE analysis based on the accessory gene content matrix. Each point represents a strain, colored according to its clade. Isolates from different clades were largely intermixed, showing no distinct clade-specific clustering. (**B**) Presence/absence heatmap of accessory genes (filtered to those present in >10% and <100% of isolates), ordered according to the core genome phylogeny.

To explore the genetic variation among different clades of the ST188 lineage, we conducted a comprehensive pan-genome analysis. As shown in Fig. 5A t-distributed stochastic neighbor embedding (t-SNE) of the accessory gene content matrix revealed no distinct clade-specific clustering. Isolates from different clades were largely intermixed and indistinguishable in the two-dimensional space, indicating that accessory genome variation shows no clear segregation by clade boundaries in the ST188 lineage. Although each ST188 clade contained some unique accessory genes, the overall proportions were very limited. Clades I–V harbored only 0–1.39% clade-specific accessory genes, whereas clades VI and VII carried slightly higher proportions (4.31% and 10.41%, respectively). This indicates that, while clade-specific accessory genes exist, their contribution to lineage diversification in ST188 is minimal. Similarly, inter-clade variation in accessory gene content was slightly greater than intra-clade variation (median ratio = 1.39), which is much lower than that observed in other epidemic lineages such as ST239 in China (median ratio = 3.85, data not shown). These findings indicate that differences in accessory gene composition among ST188 clades were relatively minimal (Fig. 5B; Table S3), with genetic diversity accumulating at the population level.

In contrast, a t-SNE analysis based on the core SNP matrix revealed clear clade-specific clustering patterns (Fig. S7A), suggesting that the evolution of the ST188 lineage is largely driven by core genome SNP variation among clades. A strict core-genome-wide association analysis using Scoary identified several clade-associated SNPs, with the number of significant SNPs ranging from 1 to 36 (Table S4). Notably, several of these clade-specific SNPs were associated with virulence-related genes, including adhesion genes (*sasH*, *sasC*, and *vWbp*), the lipase gene *lip*, and the type VII secretion system-related gene *essC*. Functional annotation of these clade-specific SNPs using the Clusters of Orthologous Groups (COG) database revealed enrichment in categories “transport and metabolism of amino acids and carbohydrates” (Fig. S7B).

## DISCUSSION

Understanding the evolutionary dynamics and dissemination mechanisms of epidemic *S. aureus* lineages is essential for mitigating their clinical impact, particularly in the context of emerging community-associated and livestock-associated strains that increasingly blur traditional epidemiological boundaries (26, 27). Although the multi-host colonizing ST188 lineage has not been described as a globally pandemic strain causing hospital-associated infections or community-associated infections (28), the 808 ST188 isolates in this study were collected from 24 countries across five continents, including even geographically isolated regions such as the Samoa islands and spanning the years from 2004 to 2023, suggesting that its global spread may have been significantly underestimated. Furthermore, a comprehensive understanding of the genomic architecture and evolutionary trajectory of ST188 remains lacking. In-depth characterization of its genomic epidemiology and dissemination dynamics is critical for effective infection control. Within this context, our study provides a comprehensive genomic and evolutionary framework of 808 ST188 isolates from a broad global collection.

Our phylogenomic analysis showed that the global ST188 lineage has diverged into seven distinct clades, with Chinese isolates primarily confined to two major clades: clades I (comprising 100% Chinese strains) and VII (71.20% Chinese strains). Additional Chinese isolates were sporadically distributed in clades III and VI, indicating multiple independent introductions into China followed by local expansions and underscoring the considerable genetic heterogeneity among Chinese ST188 strains. Notably, a recent genomic study of the endemic ST88 lineage also revealed multiple phylogenetically distinct clades undergoing independent evolutionary trajectories and clonal expansion within China (29). Further analysis showed that ST188 isolates from different countries were closely clustered in various clades (except for clade I), indicating frequent international and even intercontinental transmission. Consistently, within China, ST188 isolates from different provinces were interspersed throughout the phylogenetic tree, suggesting widespread geographic dissemination with frequent cross-regional movement. In addition, Wang et al. (8) reported that ST188 was a major MSSA lineage responsible for infections in both human (pediatric/adult patients) and in bovine hosts in Shanghai, with genomic analyses showing minimal differences between human- and livestock-derived ST188. In line with this, our study also found that non-human ST188 isolates were phylogenetically intermixed with human-derived isolates, indicating ongoing cross-host transmission. Pairwise SNP distance analysis further supported the occurrence of frequent clonal transmission events (*n* = 78) involving multiple countries and host species, underscoring the emergence of ST188 as a globally disseminated host-generalist lineage. These findings highlight the urgent need for coordinated genomic surveillance, enhanced one health-based infection control, and improved cross-sectoral data sharing. Genomic surveillance enables early detection of emerging high-risk ST188 subclades, monitors transmission across geographic regions and host species, and identifies the acquisition of critical resistance or virulence elements (e.g., SCC*mec* and prophages). These insights are essential for informing targeted infection control strategies and limiting the further dissemination of ST188 across human, animal, and environmental reservoirs.

Previous reports have identified ST188 as a predominant MSSA lineage in China, with sporadic MRSA documented in clinical settings (13, 30). However, the evolutionary origins and genomic adaptations of these MRSA derivatives have not been systematically characterized. In this study, time-calibrated phylogenomic reconstruction indicated that the ST188 MRSA isolates emerged predominantly as a single sublineage within clade VI around 1996. Notably, these MRSA isolates were phylogenetically closely related to basal MSSA strains within the same clade, suggesting that the ST188 MRSA lineage likely arose from methicillin-susceptible ancestors through the single acquisition of the SCC*mec* IVa element. This scenario is consistent with the established model in which MRSA clones independently emerge from successful MSSA backgrounds upon gaining SCC*mec*, with global evolutionary studies estimating at least ~20 independent SCC*mec* acquisition events across various MSSA lineages (31). Importantly, the ST188 MRSA sublineage and its closely related MSSA counterparts also exclusively acquired the transposon Tn*6636*, which harbors multiple resistance genes, including *erm*(B) (macrolide resistance), *aac(6′)-aph(2″)* (aminoglycoside resistance), and *dfrE* (trimethoprim resistance), along with fluoroquinolone-resistance-associated mutations *grlA* S80F and *gyrA* S84L. This accumulation of resistance determinants likely conferred enhanced selective advantage under antibiotic pressure in healthcare settings, facilitating the expansion and persistence of this sublineage in nosocomial environments. In fact, such resistance gene acquisition pattern parallels evolutionary trajectories observed in other successful hospital-adapted lineages, such as clonal complex 22 (CC22), where multiple independent acquisitions of SCC*mec* and accessory resistance genes have driven the rise of dominant MRSA variants (32).

Numerous studies have demonstrated that the acquisition of AMR genes and resistance-conferring mutations often imposes fitness costs on bacteria (33, 34). To balance resistance and host adaptation, bacteria frequently undergo trade-offs between antibiotic resistance and the expression of other fitness-related traits. Clinical MRSA strains, in particular, have been observed to alleviate such costs by downregulating or mutating energy-intensive virulence functions. For instance, hospital-associated MRSA carrying the large SCC*mec* type II element displayed attenuated toxin production and reduced growth rates compared to MSSA, while community-associated MRSA strains with the smaller SCC*mec* type IV retained higher virulence (35). Similarly, Côrtes et al. reported that ST1-SCC*mec* IV isolates from Brazil adapted to hospital environments by selectively shedding virulence factors to offset the biological costs of retaining multidrug resistance determinants (36). In line with these findings, we observed that ST188 MRSA isolates within clade VI exhibited exclusive loss of the *splD* and *splE* genes encoding serine proteases. These enzymes, encoded within the νSaβ genomic island, have been implicated in modulating *S. aureus* pathogenesis through degradation of host substrates such as mucin-16, thereby promoting tissue colonization and bacterial dissemination in pneumonia models (37). Loss of these proteases may thus represent a functional trade-off associated with adaptation to nosocomial settings. Notably, clade VI MRSA isolates also harbored a 136-amino-acid internal deletion in the *sraP* gene, which encodes a serine-rich surface glycoprotein known to mediate adhesion and epithelial invasion through receptor-ligand interactions (38). This truncation likely impairs adhesive capacity, a hypothesis supported by our preliminary phenotypic assays: clade VI isolates carrying the truncated *sraP* allele exhibited significantly reduced nasal colonization in mice and decreased adhesion to human alveolar epithelial cells, compared to isolates from other clades. Together, these genotypic changes suggest that the evolution of ST188 MRSA within clade VI involved not only the acquisition of multidrug resistance elements such as SCC*mec* IVa and Tn*6636*, but also a concomitant streamlining of virulence functions. Such trade-offs may enhance fitness under healthcare-associated selective pressures and facilitate persistence in nosocomial environments.

The mosaic distribution of MGEs across ST188 clades suggests their contributory role in shaping clade-specific phenotypes, with the Chinese subclade of clade VII particularly enriched for SaPIs and prophages. The consistent presence and similar genomic configurations of *seb*-harboring SaPI1 and SaPI2 among these Chinese subclade isolates imply vertical transmission within this lineage. In contrast, although SaPI3, φSa5, φSa7, and the novel prophage φST188-1 (which lacks known virulence genes) were detected at lower overall frequencies, their scattered presence throughout the Chinese subclade of clade VII suggests independent acquisition through convergent evolution, reflecting lineage-specific adaptive events. These findings underscore the complexity and dynamic nature of MGE integration and evolution in the ST188 genome. Notably, φSa2 family prophages in clade VII frequently integrated at multiple noncanonical chromosomal loci (most commonly between *terC* and *htrA*), potentially indicating lineage-specific recombination events or selective pressures preserving canonical insertion sites. Similar integration patterns have been reported in other *S. aureus* lineages adapting to diverse host or environmental contexts (39). Therefore, the clade-specific MGE profiles observed here highlight how horizontal gene transfer, particularly through the acquisition of MGEs, can superimpose additional variability onto the clonal framework of ST188 and give rise to lineage-specific characteristics. However, as emphasized by previous studies, the MGE repertoire in *S. aureus* is highly dynamic and often reflects recent local selective pressures rather than long-term evolutionary processes (40).

Despite the influence of MGEs on clade-specific phenotypes, our findings suggest that the evolutionary diversification of the ST188 lineage is largely driven by mutation and selection within a clonal framework, with limited but notable contributions from horizontal gene transfer, as seen in other endemic *S. aureus* lineages such as ST59 and ST88 (29, 41).

Finally, we acknowledge several limitations of this study. First, although our data set is large and globally sourced, it is not free from sampling bias. Nearly half of the genomes originate from East Asia, particularly China, which may lead to an overrepresentation of ST188 diversity in that region compared to under-sampled areas. Second, our inferences regarding cross-regional and cross-host transmission are based on phylogenetic relatedness and available metadata. Without supporting epidemiological investigations, we cannot determine the directionality or precise frequency of these transmission events. In addition, the small number of non-human isolates, together with the absence of bovine isolates, limits our ability to comprehensively evaluate cross-host transmission events and to determine whether host-specific clades or sub-clades exist within ST188. Third, while we identified specific genetic variations (e.g., *sraP* truncation) potentially associated with phenotypic traits such as reduced adhesion or colonization, these associations are based on limited experimental validation. In this study, only a small number of isolates from clades I, VI, and VII were subjected to preliminary *in vivo* and *in vitro* assays. Future studies should include a broader representation of isolates from all clades to robustly determine how specific genomic features influence the virulence and adaptive traits of ST188. In addition, functional validation using targeted genetic manipulation, such as gene deletion and complementation assays, will be crucial to confirm the causal roles of candidate genes and SNPs in mediating these phenotypic differences.

In conclusion, this study presents the first comprehensive genomic characterization of the globally distributed *S. aureus* ST188 lineage. We show that ST188 has diverged into multiple clades with evidence of independent expansions in China, particularly within clades I and VII. Frequent cross-regional, international, and inter-host transmissions were identified, supporting the emergence of ST188 as a widely disseminated host-generalist lineage. The ST188 MRSA subclade likely originated from a single SCC*mec* IVa acquisition event and showed co-acquisition of multiple resistance determinants alongside loss or truncation of key virulence genes, suggesting adaptive trade-offs under hospital-associated selective pressures. Despite clade-specific MGE patterns such as vertical inheritance of SaPIs and restricted prophage integration in clade VII, the overall evolution of ST188 appears to be primarily driven by core genome SNP variation. These findings highlight the role of gradual mutation, alongside selective MGE acquisition, in shaping the adaptation and diversification of the ST188 lineage.

## Data Availability

The whole-genome sequences of 29 ST188 *S. aureus* isolates from our collection have been deposited in the GenBank database under BioProject accession no. PRJNA1271706.

## SUPPLEMENTAL MATERIAL

Supplemental Figures msystems.00848-25-s0001.pdf Figures S1-S7.

Table S1 msystems.00848-25-s0002.xlsx Information of 808 ST188 isolates analyzed in this study.

Table S2 msystems.00848-25-s0003.xlsx Antimicrobial susceptibility of 29 ST188 isolates from our collectiona.

Table S3 msystems.00848-25-s0004.xlsx Accessory genes filtered to those present in >10% but <100% of ST188 isolates.

Table S4 msystems.00848-25-s0005.xlsx Clade-specific SNPs as identified by Scoary.

ASM does not own the copyrights to Supplemental Material that may be linked to, or accessed through, an article. The authors have granted ASM a non-exclusive, world-wide license to publish the Supplemental Material files. Please contact the corresponding author directly for reuse.
