# Supplementary material for: Clade-specific adaptation and global spread of Staphylococcus aureus ST188 with emergence of a multidrug-resistant MRSA sublineage
Source: mSystems. 2025 Sep 24;11(1):e00848-25. doi: 10.1128/msystems.00848-25 (PMC12817943; doi:10.1128/msystems.00848-25)
Supplement: Supplemental Figures — Figures S1-S7. [file msystems.00848-25-s0001.pdf]

Clade-Specific Adaptation and Global Spread of *Staphylococcus aureus* ST188 with Emergence of a Multidrug-Resistant MRSA Sublineage

Supplementary Figures

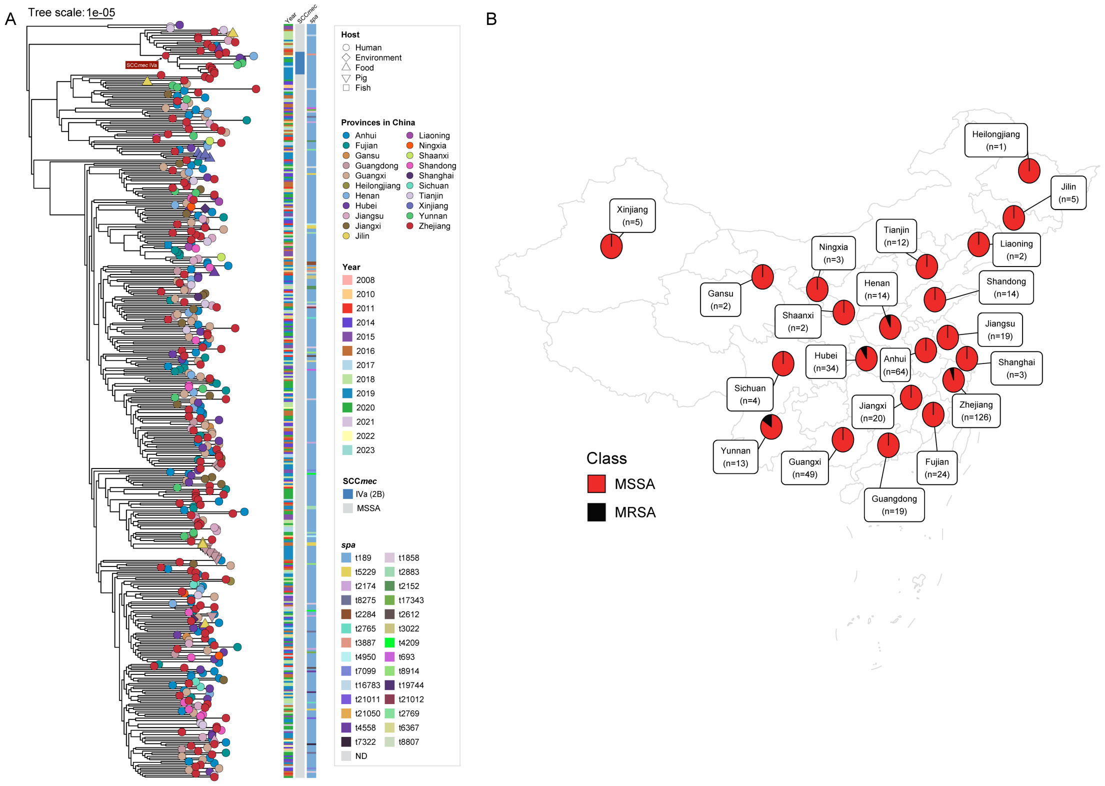

Figure S1. Relationships among 435 Chinese ST188 isolates in this study. (A) Phylogenetic tree of the 435 ST188 isolates from China, with tip colored by geographic origin and shaped according to host source. (B) Geographic distribution of the isolates, with MSSA and MRSA indicated by red and black partitions within each circle, respectively.

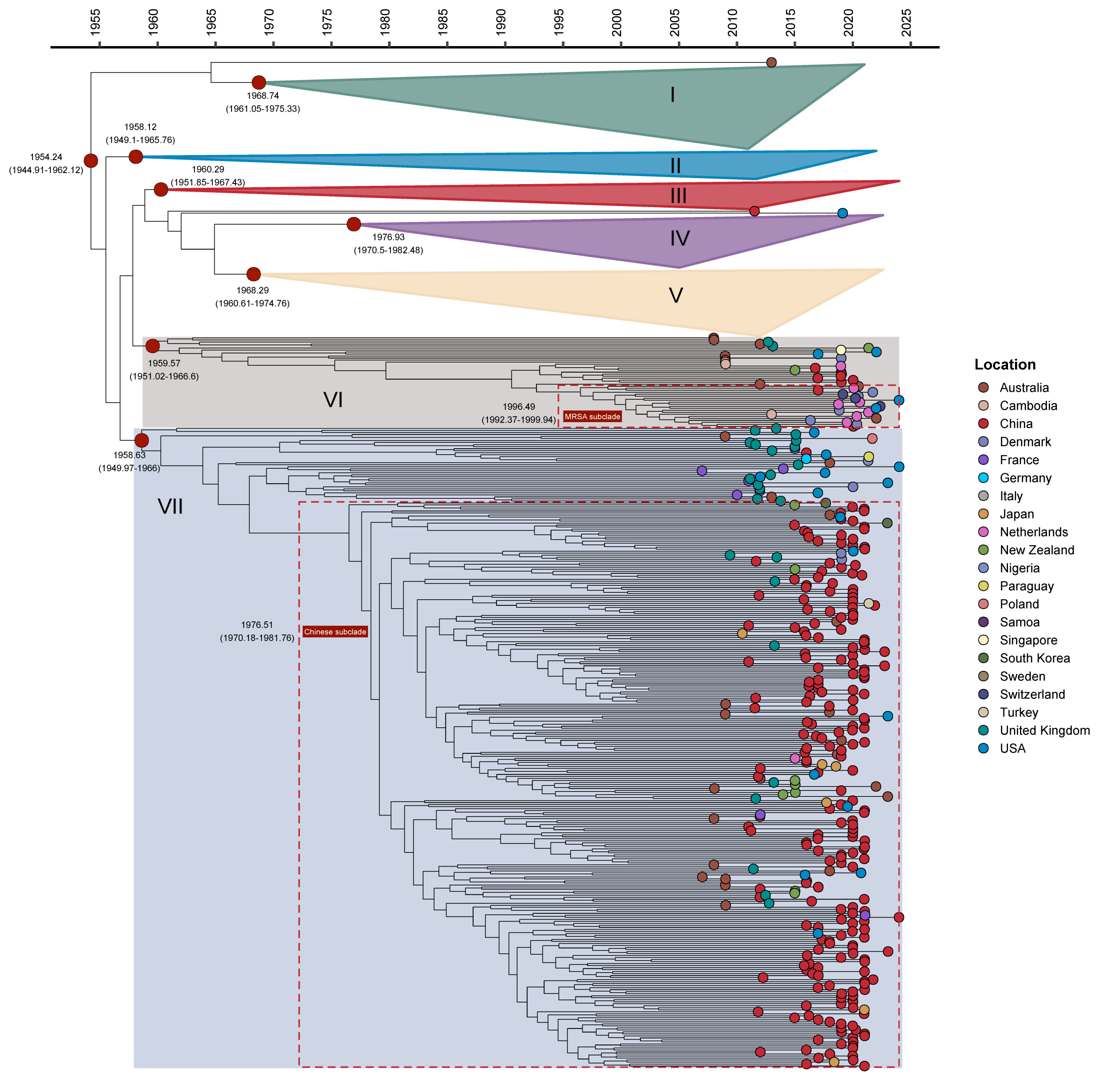

Figure S2. A dated genome-wide SNP phylogeny of ST188 isolates. The divergence time and 95% HPD intervals of main clades are shown at the respective nodes. The ST188-MRSA isolates from clade VI and strains within Chinese subclade of VII are indicated by red boxes, with relevant divergence time and 95% HPD intervals shown nearby the red boxes.

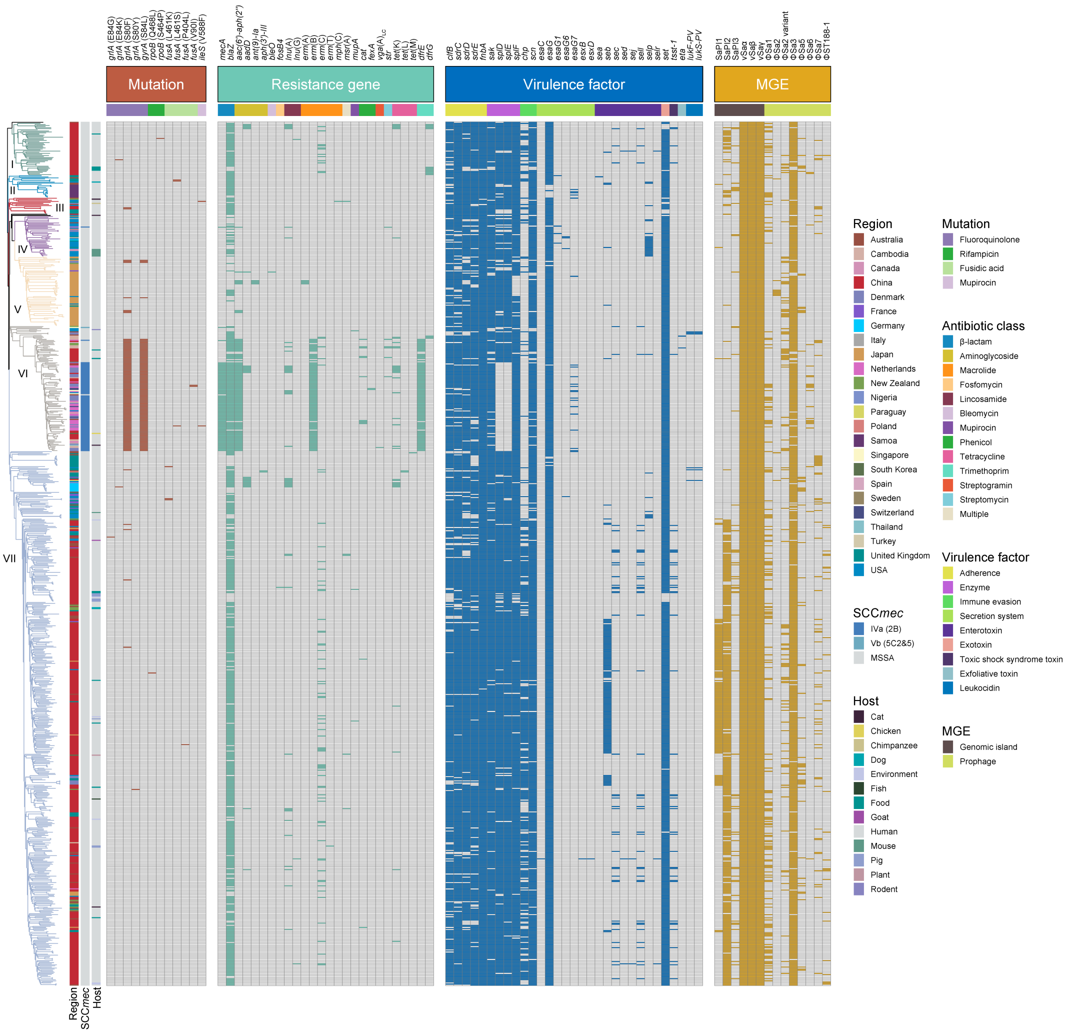

Figure S3. Distribution of intact AMR genes, virulence genes and MGEs in ST188 isolates. Heatmap showing the distribution of intact chromosomal mutations, AMR genes, virulence genes, and MGEs across ST188 isolates. Presence of each element is represented by color-coded boxes. Genes present in >90% of isolates across all clades were omitted to highlight differentially prevalent virulence traits.

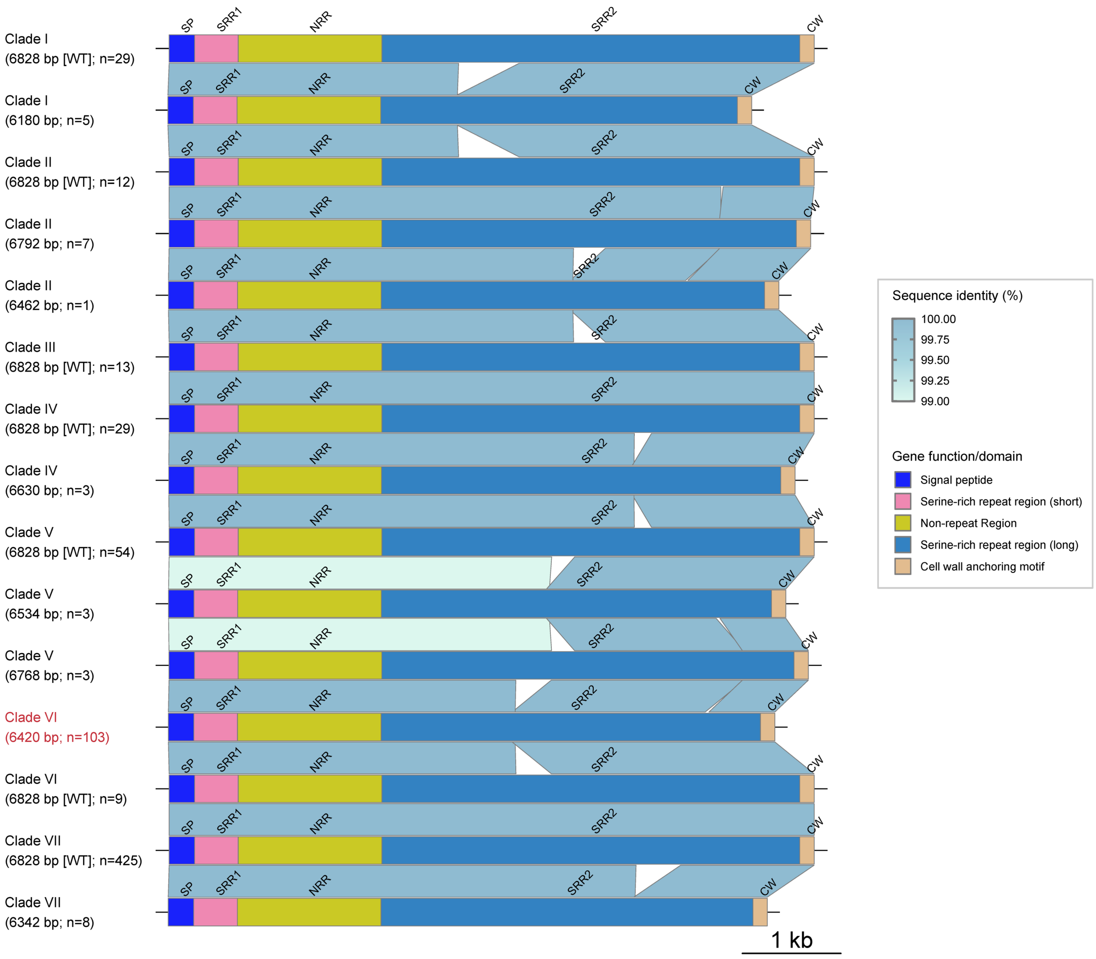

Figure S4. Comparison of *sraP* between ST188 strains in each clade. Genes are indicated by blocks and colored based on domain function classification.

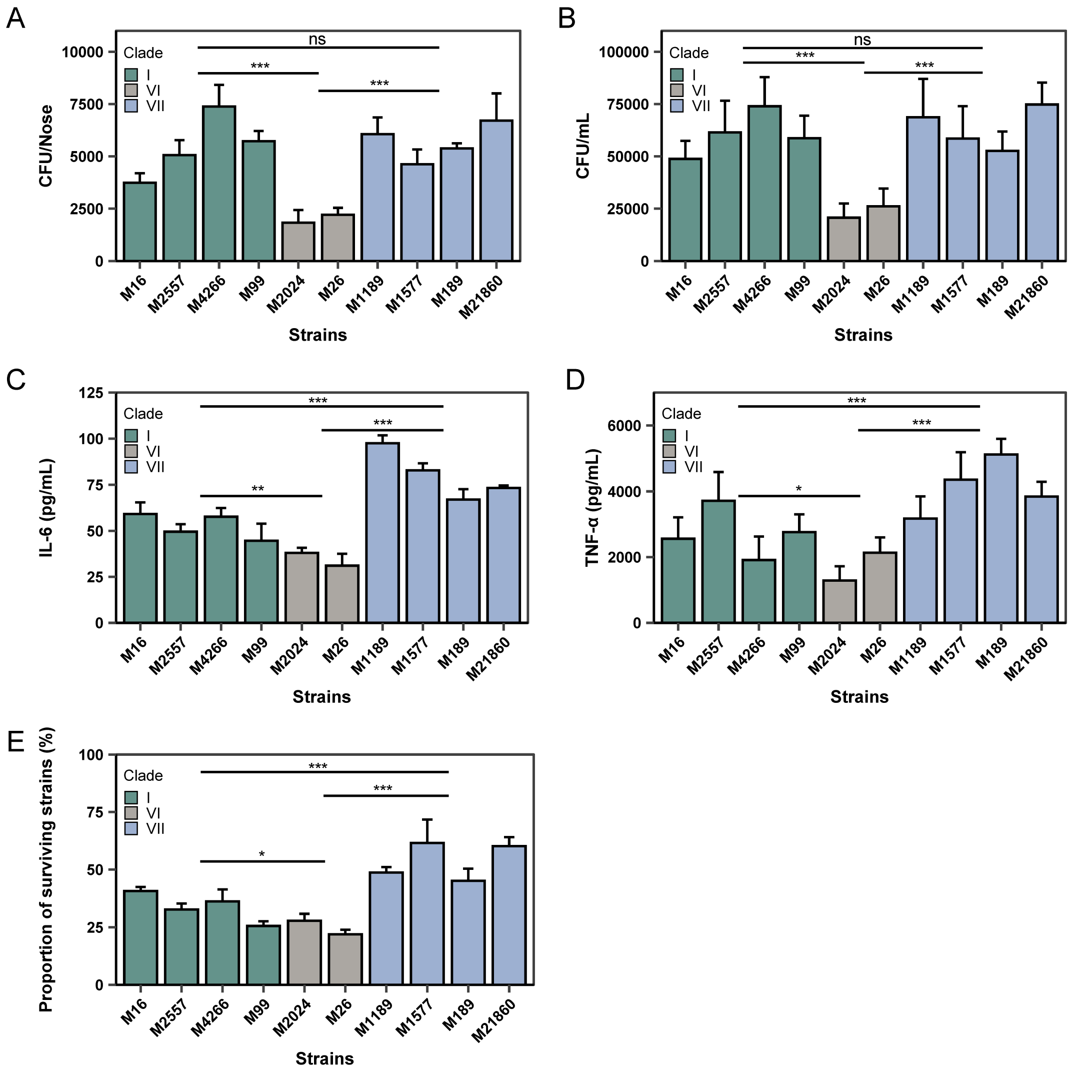

Figure S5. Comparison of virulence among different clades of ST188 strains. (A) Comparison of nasal colonization capacity of different ST188 clades. (B) Comparison of adhesive capacity in A549 human alveolar epithelial cells of different ST188 clades. (C) Comparison of ability of different ST188 clades to induce expression of IL-6. (D) Comparison of ability of different ST188 clades to induce expression of TNF-α. (E) Comparison of bacterial survival in human whole blood between different ST188 clades. *, $p < 0.05$; **, $p < 0.01$; ***, $p < 0.001$; ns, no significant difference between groups.

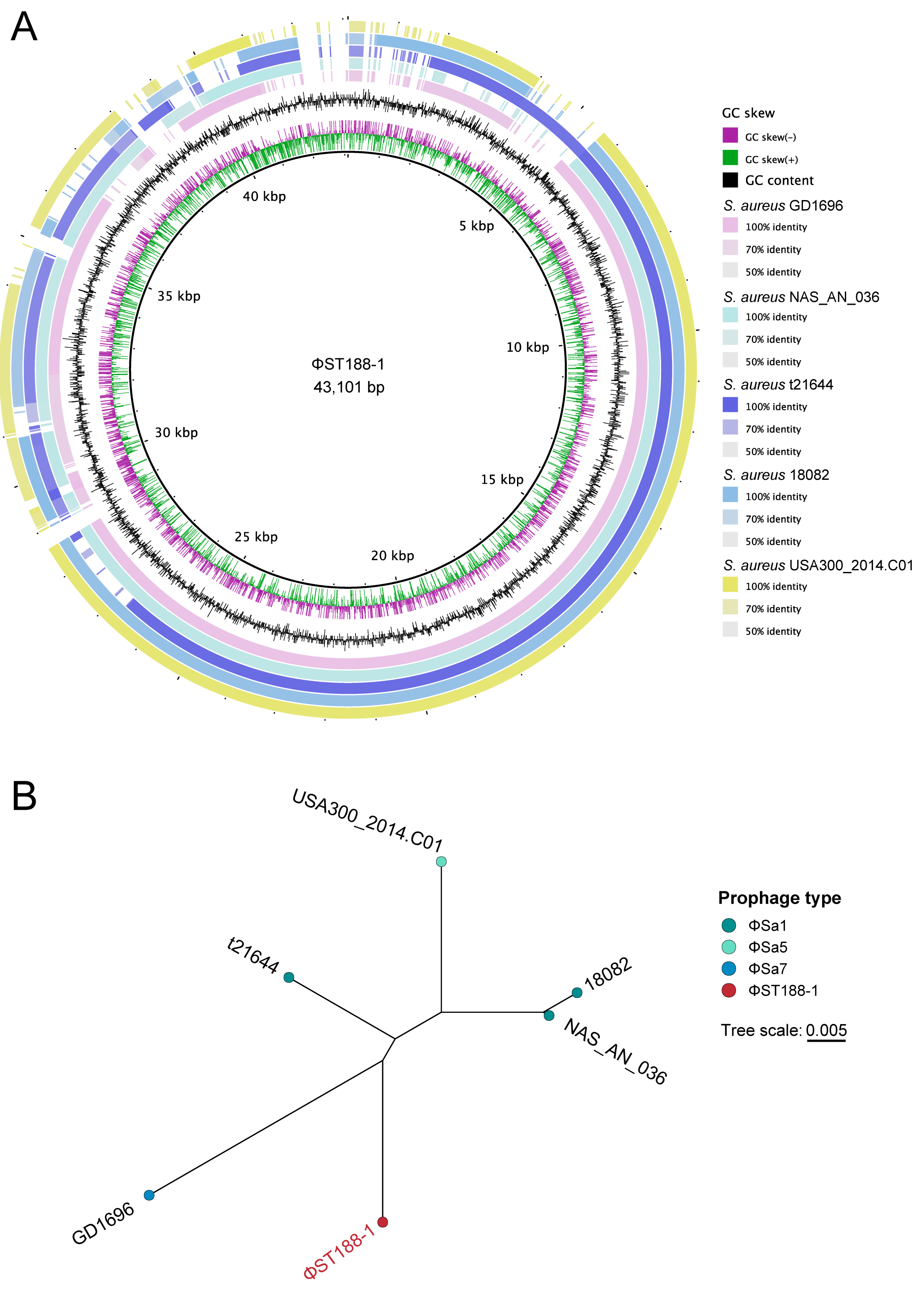

Figure S6. Schematic representation of φST188-1 and its comparison with similar prophages. (A) Circular representation of the φST188-1 and comparative genomic analysis with other similar prophages. Circles 1-5 refer (from outer to inner circle) to homologous regions of selected *S. aureus* relative to the φST188-1. Circles 6-7

represent GC content and GC skew of the φST188-1, respectively. (B) Phylogenetic analysis of the φST188-1 with other similar prophages, with tip colors representing the prophage types carried by the strains.

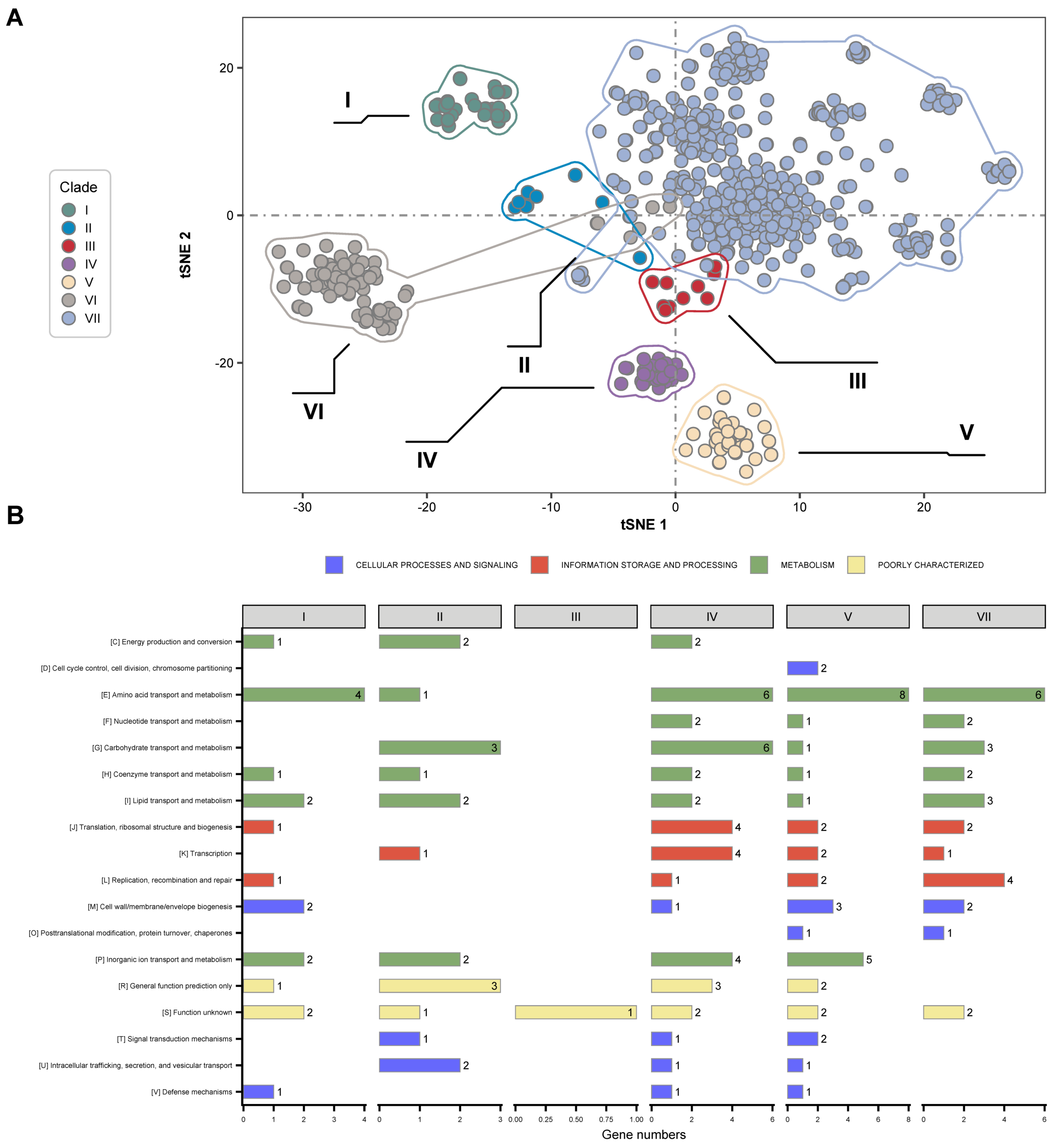

Figure S7. Core SNP diversity among ST188 clades. (A) t-SNE analysis based on the core SNP matrix of 808 isolates, where each circle represents an isolate, colored according to its clade. Clear clade-specific clustering was observed, indicating that core-genome SNP variation strongly differentiates clades within the ST188 lineage. (B) Functional classification of genes containing clade-specific SNPs. The bar plots are grouped by clade, with each plot showing the number of affected genes (x-axis) per COG functional category (y-axis). Colored bars correspond to major COG functional classes. Clade-specific SNPs were most frequently located in genes involved in amino

acid and carbohydrate transport and metabolism.
